# Close is clever! Spatial proximity shapes expertise attribution

**DOI:** 10.3389/fpsyg.2026.1763841

**Published:** 2026-08-24

**Authors:** Scarlett Friedrich, Roman Linne

**Affiliations:** Department of Social Psychology, Faculty of Humanities and Social Sciences, Helmut Schmidt University, Hamburg, Germany

**Keywords:** spatial proximity, expertise, person perception, social cognition, persuasion

## Abstract

Individuals infer expertise from spatial proximity: the closer a person is to an object, the more expertise they are presumed to have. In four experiments (total *N* = 606) we investigated the relationship between spatial proximity and attributed domain-specific expertise, as well as its influence on persuasion. Study 1 indicated that participants attributed greater expertise to individuals depicted with (vs. without) domain-relevant objects. Study 2 showed that individuals positioned proximally (vs. distally) to objects were ascribed more expertise. Study 3 revealed a bidirectional relationship: participants placed domain-relevant objects closer to individuals described as experts in the corresponding domains. Study 4 demonstrated that spatial proximity also affects persuasion. A communicator positioned proximally (vs. distally) to the attitude object elicited stronger attitude change. Together, these findings provide first empirical evidence that spatial proximity influences perceived expertise, highlighting the relevance of spatial cues in person perception and persuasion.

## 1 Introduction

Occasionally, people encounter situations in which they lack the knowledge or skills needed to make a judgment or complete a task. Such situations range from abstract domains (e.g., evaluating political conditions in a distant region) to more tangible, practical challenges (e.g., repairing a malfunctioning car). In these contexts, it is reasonable to seek advice from someone with relevant *expertise*. To identify such *experts*, people often rely on contextual or perceptual cues, such as academic titles or occupational attire. In the present research, we investigate whether the perception of a person’s spatial proximity to a relevant object also serves as a cue to expertise.

An expert is typically defined as someone who possesses more knowledge and outperforms most others in a given domain (Ericsson and Charness, 1997). Domains may include professional fields (e.g., architecture) or hobbies (e.g., calligraphy; Chi, 2006). Compared to laypersons, experts show superior memory (e.g., Chase and Simon, 1973; de Groot, 1978), task performance (e.g., Jarodzka et al., 2010; Sheridan and Reingold, 2014), and problem solving (e.g., de Groot, 1978; Hershey et al., 1990; Jarodzka et al., 2010).

Given the extensive knowledge and skills of experts (Ericsson and Charness, 1997), it is often reasonable to consult one when seeking reliable information or assistance. Like many other socially meaningful characteristics (e.g., sociability or trustworthiness), expertise may also be the result of an attribution process (Gobet, 2016). When objective information about a person’s knowledge or performance is not available, individuals may similarly rely on such cues to infer expertise. These may include academic titles, distinctive clothing (e.g., a laboratory coat or a boiler suit), perceived quality of actions and statements, and more abstract communication styles (e.g., Chaiken and Maheswaran, 1994; Erb et al., 2007; Mieg and Evetts, 2018; Petty et al., 1981; Reyt et al., 2016).

More generally, when forming judgments about another person (hereinafter “target”), individuals often rely on available cues in the environment and abstract from the information provided in a situation. For example, individuals form impressions of a target’s openness to new experiences or extraversion based on the furnishings of a bedroom (Gosling et al., 2002), clothing (Naumann et al., 2009), or music preferences (Rentfrow and Gosling, 2006). Experimental evidence further shows that observers draw conclusions about a target’s characteristics, such as competency, based on objects in close proximity to the target (e.g., expensive- vs. non-expensive-looking clothing; Oh et al., 2020). Altogether, this research suggests that objects in a target’s environment serve as cues from which observers infer attributes.

Objects in close spatial proximity to a target may constitute such a cue. In social perception, spatial relations are often interpreted as meaningful rather than incidental. For example, interpersonal distance is commonly used to infer the relationship between individuals, with closer proximity signaling greater familiarity or relatedness (Hall, 1969, 1973; Little, 1965; Wellens and Goldberg, 1978). Extending this logic, spatial proximity between a target and an object may likewise signal a meaningful relation between the two. In the present research, we focus on the spatial distance observers perceive between a person and an object, which we refer to as *non-egocentric distance*. This type of distance perception, and the inferences they support regarding the relationship between the two entities, do not necessarily depend on self-referential psychological distance. Prior work further suggests that spatial proximity between entities can serve as a cue when evaluating abstract properties, such as the effectiveness of a product (Chae et al., 2013). Building on this, we propose that a person’s spatial proximity to an object representing a particular domain can serve as an additional cue to their expertise. Hereinafter, we refer to such objects as “relevant objects.”

We consider expertise to be inferred from spatial proximity. Titles, attire, and so forth are valid cues that signal expertise, but they are limited to specific domains and often require culture-specific knowledge to be interpreted meaningfully. For example, individuals can only construe a doctoral degree as an indicator of expertise if they understand its social meaning. Moreover, expertise in many domains is not communicated through formal credentials or distinctive occupational attire. Florists, for instance, typically do not hold university degrees, and programmers often lack visually distinctive work clothing. In contrast, spatial proximity between a person and an object may serve as a more broadly applicable cue to expertise. Assuming that expertise can be attributed based on spatial proximity, such judgments may resemble other forms of cue-based inference in person perception and object evaluation (e.g., Chae et al., 2013; Gosling et al., 2002; Naumann et al., 2009).

We propose that the attribution of expertise based on spatial proximity follows a two-step process. First, observers assign a target to a meaningful category activated by an object in the immediate surroundings (Allport, 1954; Bodenhausen and Macrae, 1998; Fiske and Neuberg, 1990). In a given situation, this category corresponds to the domain of expertise represented by the object. Highly accessible category-related information is likely to influence subsequent impressions and evaluations of the target (Bargh et al., 1986; Decoster and Claypool, 2004; Higgins and Kruglanski, 1996). Categorizing a target based on a nearby object may also lead observers to draw spontaneous inferences about the target (Gilbert, 1989; Trope and Alfieri, 1997). Second, observers use spatial proximity to infer the relationship between the target and the object. Contextual information may shape the interpretation of social information by rendering certain inferences more coherent or plausible than others (e.g., Bless and Schwarz, 2010). A target depicted in close proximity to an object may be perceived as more strongly associated with, and more familiar with, the domain represented by that object (Chae et al., 2013; Hall, 1969, 1973), which may make one inference from the first step more likely than others. Consequently, observers may attribute expertise to the target within the related domain.

Expertise judgments based on spatial proximity may arise from different inferential processes. In some situations, observers may rely on relatively intuitive reasoning processes, particularly when they lack either the motivation or the background knowledge to engage in more elaborate processing (Chae et al., 2013). Alternatively, expertise judgments based on spatial proximity may result from more deliberate, propositional reasoning when individuals are motivated to engage in effortful processing (Chaiken et al., 1989; Petty and Cacioppo, 1986). This process may include inferences about the relationship between a person and an object or the reason for their close proximity (cf. de Houwer, 2018; Gawronski and Bodenhausen, 2018). For illustration, an object may activate the category “cars” which leads observers to reason that the target is knowledgeable about and familiar with this specific object. This inferred expertise may then generalize to the broader domain represented by the object (i.e., cars).

The present research comprises four experiments investigating how spatial proximity informs judgments of expertise. We propose that people attribute more expertise to targets closer to domain-relevant objects and, in turn, position targets closer to relevant objects when they believe those targets have expertise. Across four studies, we test these predictions and assess their implications for persuasion. Study 1 investigates whether objects in a target’s vicinity serve as cues to expertise. Study 2 tests whether expertise attributions vary systematically with the degree of spatial proximity. Study 3 examines the bidirectionality of the relationship, investigating whether perceived expertise shapes the spatial positioning of targets relative to relevant objects. Study 4 extends these findings to a persuasion context, assessing how spatial proximity influences attitude formation. Together, these studies provide converging evidence that spatial proximity serves as a diagnostic cue for expertise attribution and carries meaningful consequences for persuasion.

All studies reported in this manuscript were approved by the Ethics Committee of Psychology at the Faculty of Humanities and Social Sciences, Helmut Schmidt University. Participants in all experiments were required to be fluent in German and to have no prior exposure to our research. In Studies 2 and 3, participation was restricted to desktop or laptop computers. In Study 1 (conducted last), we applied additional criteria: Participants had to hold German nationality and reside in Germany to ensure data quality. The order in which the studies are described does not correspond to the order in which they were conducted.

## 2 Study 1

Previous research has shown that observers rely on objects in a person’s environment to draw inferences about that individual (Gosling et al., 2002; Naumann et al., 2009; Oh et al., 2020). Building on this idea, we hypothesize that targets located near relevant objects are attributed greater expertise. Study 1 tested whether individuals infer others’ domain-specific expertise from nearby objects. Participants viewed pictures of targets either in the presence of an object or alone and rated the targets’ expertise in object-related domains. We hypothesized that targets presented with objects would be ascribed greater expertise than targets presented alone. It also seems plausible that this effect may depend on the domain involved, a possibility we explored in additional analyses. The corresponding preregistration is available at https://osf.io/htzgq/overview?view_only=aa51552bc25e4efd8958a9bd6fc46363.

### 2.1 Method

#### 2.1.1 Participants and design

Of 274 participants, four were excluded because their data were incomplete, and one because the participant repeated the study after an initial failed attempt. The final sample therefore comprised *n* = 269 participants (106 female, 160 male, 3 did not specify gender; *M*_*age*_ = 36.03, *SD*_*age*_ = 11.11). We recruited participants via Prolific^1^ and compensated them with EUR 0.65.

Participants were randomly assigned to one of two conditions of a single-factor (object: present vs. absent) between–subjects design with seven target replications. A power analysis for a mixed ANOVA (using G*Power 3.1; Faul et al., 2007) with object as a between–subjects factor and trial (Targets 1 to 7) as a within–subjects variable, indicated that *N* = 266 participants were required to achieve a power (1–β) of 0.90 for detecting a medium effect of *f* = 0.20 at an alpha level of 0.05. To allow for attrition, we aimed for a total sample of *N* = 270.

#### 2.1.2 Procedure and dependent variable

Participants were recruited for a study examining person perception. They were informed that they would view pictures of different people and evaluate each person based on their impressions. We encouraged participants to rely on their intuition when forming these judgments.

The experiment consisted of seven trials, each including an image of a target and six statements about that target. The order of trials was randomized for each participant. Depending on the experimental condition, the images either depicted the target alone (object absent) or the target together with a nearby object (object present; e.g., a construction plan). At the beginning of each trial, the image was presented for 7 s. After this interval, six statements appeared sequentially below the image (i.e., “This person is knowledgeable about architecture.”), which participants used to evaluate the target. After selecting a response on a 7-point Likert scale ranging from 1 (*strongly disagree*) to 7 (*strongly agree*), the next statement appeared automatically. After participants had responded to all six statements, the study automatically continued with the next trial (an example of the stimulus material is provided in the Supplementary material).

Of the six statements, one was identical across all targets (i.e., “The person is trustworthy.”), two represented personality traits that were randomly assigned to targets before the experiment (e.g., conscientiousness, humorous), and the remaining three captured perceived expertise in three distinct domains. The first three statements were included primarily to maintain the impression that the study concerned person perception.

The same domains were used to assess the target’s expertise across both levels of object presence (present vs. absent). One domain corresponded to the object shown in the object-present condition (e.g., “The person is knowledgeable about architecture”). Although only participants in the object-present condition viewed the object (e.g., the construction plan), participants in both conditions evaluated the target’s expertise in this domain. The second domain was conceptually related to the object (e.g., “urban planning”), and the third domain was unrelated (e.g., “marine animals”). At the end of the experiment, participants were thanked and debriefed.

### 2.2 Results and discussion

To test the hypothesis that objects in spatial proximity to targets serve as cues from which observers deduce the targets’ expertise, we conducted a mixed ANOVA with object (present vs. absent) as a between–subjects factor, trial (Targets 1 to 7) as a within–subjects variable, and expertise ratings as the dependent variable. To maintain consistency across studies and ensure a conservative test of significance, degrees of freedom for within–subject effects were adjusted using the lower-bound correction. Most importantly, the analysis revealed a significant main effect of object, *F*(1, 267) = 279.67, *p* < 0.001, η^2^ = 0.51. Additionally, we found a significant main effect of trial, *F*(6, 267) = 66.24, *p* < 0.001, η^2^ = 0.20, as well as a significant object × trial interaction, *F*(6, 267) = 42.70, *p* < 0.001, η^2^ = 0.14.

The main effect of object supports our hypothesis that participants relied on objects in targets’ spatial proximity to infer their expertise. As expected, targets presented with objects were rated higher in expertise (*M* = 5.84, *SD* = 0.70) than those presented alone (*M* = 4.36, *SD* = 0.10). The main effect of trial indicates that perceived expertise differed across stimuli, which is unsurprising, because participants reasonably evaluate different expertise domains differently (e.g., architecture vs. bicycles). Finally, the interaction implies that the influence of objects on expertise judgments varied across trials, suggesting that some targets’ attributions were more strongly affected by nearby objects than others.

Follow-up independent-samples *t*-tests for each trial revealed significant effects of object presence across all stimuli (all *p* < 0.001; see Supplementary material for full results). Effect sizes varied across domains, with the largest effects observed for skateboards, guitars, and dogs, and smaller effects for golfing and bicycles. This pattern suggests that while participants reliably infer expertise from nearby objects, the magnitude of this inference depends on the domain.

Exploratively, we examined whether the proximity–expertise effect is attenuated for “everyday expertise.” This analysis was motivated by the assumption that expertise attributions may differ depending on whether a domain is perceived as requiring specialized versus more common knowledge. For this, we selected two example targets from the seven trials: a common expertise (bicycles) and a special expertise (architecture). We conducted a mixed ANOVA with object (present vs. absent) as between–subjects factor and expertise type (common vs. special) as within–subjects variable on expertise ratings. The analysis showed a significant object × expertise type interaction, *F*(1, 267) = 7.08, *p* = 0.008, η^2^ = 0.03. Moreover, it showed significant main effects for both, object, *F*(1, 267) = 74.48, *p* < 0.001, η^2^ = 0.22, and expertise type, *F*(1, 267) = 9.22, *p* = 0.003, η^2^ = 0.03. For both domains, perceived expertise was higher when the relevant object was present than absent (bicycles: *M*_*pres*_ = 5.55, *SD*_*pres*_ = 1.01 vs. *M*_*abs*_ = 4.85, *SD*_*abs*_ = 1.26; architecture: *M*_*pres*_ = 6.01, *SD*_*pres*_ = 1.02 vs. *M*_*abs*_ = 4.88, *SD*_*abs*_ = 1.08). Notably, the effect of object presence was larger for special than for common expertise, indicating an attenuation of the effect in the common expertise condition, consistent with the significant interaction. These results indicate the effect was larger for the special-expertise target than for the common-expertise target.

Finally, we explored whether expertise in domains belonging to the same superordinate category as the depicted domain (e.g., dogs and cats, both mammals) would be rated higher than expertise in unrelated domains (e.g., gardening), reflecting a general assumption of category-based generalization. To test this, we conducted a mixed ANOVA on expertise ratings with trial (Targets 1 to 7) and domain type (depicted, related, unrelated) as within–subjects variables, and object (present vs. absent) as a between–subjects factor. The ANOVA revealed a significant domain type × object interaction, *F*(2, 267) = 230.47, *p* < 0.001, η^2^ = 0.46. When objects were present, expertise ratings dropped sharply from depicted to both the related and unrelated domains, which did not differ significantly (full details and pairwise comparisons are reported in the Supplementary material). The interaction between domain type and object further revealed that the presence of objects particularly increased expertise ratings for depicted domains relative to related and unrelated domains. The results of the contrast analyses and pairwise comparisons showed a clear ordering of expertise ratings: participants attributed the highest expertise to the represented domain, lower expertise to related domains, and the lowest expertise to unrelated domains.

Study 1 demonstrates that individuals use objects in another person’s spatial proximity to infer that person’s expertise in the relevant domain. Across seven trials, participants attributed greater corresponding expertise to targets presented next to objects representing specific domains than to targets presented without these objects. Our exploratory analyses suggest that this effect is stronger for expertise domains requiring specialized knowledge or formal training, whereas it is weaker for everyday skills, such as riding a bicycle, that most people acquire without formal instruction. The effect appeared to be largely confined to domains represented by an object, with expertise ratings for related and unrelated domains showing little evidence of an object-related increase.

The results of this initial experiment indicate a relatively straightforward “object-presence effect” on expertise attributions. Although this finding is conceptually unsurprising, we consider it as a necessary first step toward establishing the existence of a basic spatial proximity effect, that is, demonstrating that objects located near a person systematically shape judgments of that person’s expertise. Building on this baseline, the following experiment tests whether the effect depends specifically on objects being in close spatial proximity to the target rather than merely being present, by systematically varying the distance between targets and objects.

## 3 Study 2

Study 2 was set out to test the hypothesis that proximal (vs. distal) spatial distances lead to higher (vs. lower) expertise ratings. To that end, we presented participants with images of targets depicted either in closer or more distant proximity to an object, using relatively subtle variations in spatial distance. Participants were asked to assess each target’s expertise in relation to the respective object. We hypothesized that participants would ascribe more (vs. less) expertise to a target positioned proximally (vs. distally) to an object. The corresponding preregistration is available at https://osf.io/m583p/?view_only=984f2ef3eb30420b94c0ea44fa16e16d. Deviations from the preregistration are reported in the Supplementary materials.

### 3.1 Method

#### 3.1.1 Participants and design

The sample consisted of 115 complete data sets. The data of *n* = 17 participants were excluded due to their participation in one of our previous studies. Thus, analyses are based on a total of *n* = 98 participants (43 female, 54 male, 1 did not specify gender; *M*_*age*_ = 31.59, *SD*_*age*_ = 10.26). We recruited participants via Prolific (see Footnote 1) and compensated them with EUR 1.80. Participants were randomly assigned to the conditions of a single-factor (distance: proximal vs. distal) between–subjects design that included a stimulus replication with four different targets. The power analysis for a mixed ANOVA (using G*Power 3.1; Faul et al., 2007) with distance as a between–subjects factor and trial (Targets 1 to 4) as a within–subjects variable suggested a target sample size of 78 participants to obtain a power (1–β) of 0.80 for detecting a medium-sized effect *f* = 0.25 at a standard 0.05 alpha error probability. Assuming not all participants would complete the study, we aimed for a total sample size of at least 90.

#### 3.1.2 Procedure and dependent variable

Participants took part in an online study purporting to investigate person perception. Initially, participants learned we would provide them with pictures of different people and ask them about their impressions of these people. Then, they received two example pictures and a reiteration of the forthcoming task (for details see the Supplementary material).

Following a short practice trial, the actual experiment began, consisting of 11 trials, each trial presenting one target. Trial order was predetermined, randomized, and identical across both distance levels. Four of the trials depicted the target together with an object, a car, a robot, a barrel of nuclear waste, and a whale carcass, representing the domains of expertise “cars,” “robots,” “radioactivity,” and “marine animals.” In these critical trials, we manipulated spatial proximity by varying the physical distance between targets and objects. To display a proximal distance, targets and objects were placed right next to each other; to display a distal distance, targets and objects were placed at opposing image borders (example stimuli are provided in the Supplementary material). The remaining seven trials served as fillers to substantiate the cover story.

At the beginning of each (critical) trial, only the picture of the target was displayed for 5 s. Subsequently, eight questions appeared sequentially underneath the picture. Two of these questions pertained to the target’s *expertise* in the domain represented by the object (e.g., “How do you estimate the person’s expertise in relation to marine animals?,” 1 = *no expertise* to 7 = *high expertise*). For the remaining questions, participants were asked to rate other characteristics of the target unrelated to expertise (e.g., assumed creativity) and to report on their perceived similarity to the target. After participants had answered all questions of one trial, the study automatically proceeded with the next trial, depicting a different target. The filler trials followed the same procedure, except that the two expertise questions were replaced by two questions assessing other characteristics of the target, while the similarity question remained unchanged.

We included additional items controlling for the programmed randomization and possible participation in a previous study. At the end of the experiment, participants were thanked and debriefed.

### 3.2 Results and discussion

Initially, we averaged the two items measuring expertise separately for each critical trial to form four expertise indices (Spearman-Brown coefficients ranged from ρ = 0.89 to ρ = 0.96).

To test the hypothesis that perceived expertise is a function of distance, we conducted a mixed ANOVA with distance (proximal vs. distal) as a between–subjects factor, trial (Targets 1 to 4) as a within–subjects variable, and expertise ratings as the dependent variable. The results showed a significant main effect of distance, *F*(1, 96) = 4.09, *p* = 0.046, η^2^ = 0.04, as well as of trial, *F*(3, 288) = 9.14, *p* < 0.001, η^2^ = 0.09. The trial × distance interaction did not reach statistical significance *F*(3, 288) = 0.52, *p* = 0.672, η^2^ = 0.01.

The main effect of distance is consistent with our hypothesis, indicating that the attribution of expertise was a function of spatial proximity. On average, participants rated the targets’ expertise higher when the targets were positioned proximally to the corresponding objects (*M* = 5.28, *SD* = 1.49) compared to when they were positioned distally (*M* = 4.87, *SD* = 1.36; see Supplementary material for more details). The main effect of trial suggests that the attribution of expertise varied with the stimuli. This finding is not particularly surprising. Different expertise domains are likely evaluated differently (e.g., cars vs. robots). Since it is unrelated to perceived distance, it does not address the research question at hand. The missing of a significant interaction between trial and distance implies that spatial proximity affected expertise ratings independent of expertise domain.

Study 1 showed that individuals infer a person’s expertise from the mere presence of a relevant object. Study 2 extends this finding by demonstrating that expertise judgments are further influenced by the spatial proximity of that object. Participants attributed more expertise to targets proximally (vs. distally) to the objects. In the next study, we opted to test the hypothesis that the relationship is bidirectional.

## 4 Study 3

With Study 3, we attempted to test the scope of the relationship between spatial proximity and perceived expertise. Thus far, we have presented (preliminary) evidence for a directional relationship: Observers attribute expertise to targets located in proximity to objects associated with relevant domains. We hypothesize that this effect is driven by perceived shared category membership, which leads to inferred familiarity with the object and, in turn, to the attribution of expertise. However, we assume that this effect is bidirectional: If a target is already perceived as an expert, they should activate the corresponding category in much the same way as a representative object. Moreover, not only should spatial proximity imply familiarity and expertise, but we also anticipate that perceived familiarity and expertise increase the expectation of spatial proximity. For example, someone needing to repair a malfunctioning car would likely turn to an expert, assumed to be near the tools and equipment associated with car repair.

Crucially, our hypothesis goes beyond the notion that perceived expertise merely signals a categorical association, such as implying ownership of relevant objects. Rather, we argue that perceived expertise gives rise to spatial expectation: relevant objects are assumed to be physically close to the expert (compared to someone with expertise in an unrelated domain), not merely present in the same environment.

Participants received descriptions suggesting the targets’ expertise in various domains and were subsequently asked to arrange several objects around each target. We expected participants to position objects closer to the targets when the objects aligned with the targets’ domains of expertise. Details of the preregistration are available at https://osf.io/cfumd/?viewonly=aa13df044c3144f98798d5be6e720da4.

### 4.1 Method

#### 4.1.1 Participants and design

The data of one participant was dropped from the analyses due to premature termination of the study. Our final sample thus included a total of 120 participants (51 female, 59 male, 10 did not specify gender; *M*_*age*_ = 29.38, *SD*_*age*_ = 9.20). We recruited participants via Prolific (see Footnote 1) and paid each EUR 1.63 for their participation. Participants were randomly assigned to the conditions of a single-factor (expertise: matching vs. non-matching) between–subjects design with stimulus replication. We ran a power analysis for a mixed ANOVA (conducted with G*Power 3.1; Faul et al., 2007) with three levels of a within–subjects variable and expertise as the between-subjects factor. Aiming at a power of (1–β) of 0.90 for detecting a medium-sized effect of *f* = 0.25 at a standard 0.05 alpha error probability we needed to recruit a minimum of 116 participants.

#### 4.1.2 Procedure and dependent variable

Initially, participants learned that the alleged research question revolved around whether a person’s description allowed inferences about their home furnishings. For this purpose, participants were presented with the (self-)descriptions of three persons (i.e., the targets) and asked to furnish a room for each using a fixed set of objects, including a critical object.

Each target description was approximately 100 words long, implied expertise in a certain domain, and was accompanied by a picture of the respective target (see Supplementary material). The descriptions were pre-tested to ensure they successfully conveyed the intended domain of expertise. Depending on the condition (expertise matching vs. non-matching), targets were either introduced as a photographer, a dog trainer, and a bass guitarist (with the critical objects matching the domain of expertise) or a confectioner, a hobby gardener, and a basketball enthusiast (critical objects not matching expertise). Accordingly, the critical objects were a camera, a dog, and a bass guitar. The descriptions of each target were mostly parallel across condition levels, differing only regarding the expressions indicating the domain of expertise. For example, in the experimental condition participants read: “*[*…*] For instance, a couple of years ago I fulfilled a dream when I* ***adopted a dog.*** *[*…*] After that, I read everything I could get my hands on and tried out a lot of things. [*…*],”* whereas in the control condition, participants read: *“[*…*] For instance, a couple of years ago I fulfilled a dream when I* ***leased a garden lot***. *[*…*] After that, I read everything I could get my hands on and tried out a lot of things. [*…*].”*

After reading a description, participants saw a picture of the target standing in an empty room. Underneath the picture, participants found a set of seven objects, including the corresponding critical object (an example image can be found in the Supplementary material). The remaining objects included pieces of furniture as well as decorative objects, such as a bookcase and flowers. Prior to the main experiment, participants received detailed instructions on how to select and place objects and completed a practice trial.

We asked participants to arrange all objects in the room via drag-and-drop, according to how they believed the target would furnish it. This procedure was then repeated with two further targets and new rooms, including new critical and distractor objects. The order in which we presented the targets was randomly determined in advance of the experiment and held constant for all participants. At the end of the study, participants could report problems with the procedure, were thanked, and debriefed.

### 4.2 Results and discussion

First, we computed the Euclidian distances, measured in pixels, between each target and the corresponding critical object. Next, we submitted expertise (matching vs. non-matching the critical object) as a between–subjects factor, trial (Targets 1 to 3) as a within–subjects variable, and distance as the dependent variable to a mixed ANOVA. Since Mauchly’s test indicated a violation of sphericity for trial (*W* = 0.710, χ^2^(2) = 40.01, *p* < 0.001), we applied Huynh-Feldt corrections to the degrees of freedom (ε = 0.790; Girden, 2003). The analysis returned a significant main effect of expertise, *F*(1, 118) = 6.81, *p* = 0.010, η^2^ = 0.06 as well as for trial, *F*(1.58, 186.55) = 108.56, *p* < 0.001, η^2^ = 0.48. The trial × expertise interaction did not reach statistical significance, *F*(1.58, 186.55) = 0.28, *p* = 0.704, η^2^ = 0. The main effect of expertise indicates that, on average, participants placed the critical objects closer to targets with matching expertise (*M*_*matching*_ = 231.56, *SD*_*matching*_ = 112.61) than to those with non-matching expertise (*M*_*non–matching*_ = 265.86, *SD*_*non–matching*_ = 128.28; analyses on a trial-level can be obtained from the Supplementary material).

The main effect for trial emerged because the distances between targets and critical objects varied across the three different targets (e.g., dogs were placed closest; see Table 1). However, this effect was independent of expertise.

**TABLE 1:** Averaged Euclidian distances between target and critical object for matching and non-matching expertise.

Critical object	Expertise	
	Matching	Non-matching
Camera	335.58 (149.84)	380.30 (141.41)
Dog	97.58 (34.26)	117.28 (62.12)
Bass guitar	261.51 (153.73)	300.00 (181.31)

*N* = 120. The distances were measured in pixels, greater numbers represent greater distances. Standard deviations are in parentheses.

Going beyond the preregistered analysis, we computed an additional mixed ANOVA to test whether the expertise manipulation affected the placing of critical and distractor objects differently. Distractor objects were pieces of furniture and decorative objects unrelated to the descriptions. The analysis revealed a significant expertise × object type (critical vs. distractor) interaction, *F*(1, 236) = 7.11, *p* = 0.009, η^2^ = 0.06 (for details on the analysis, see Supplementary material). This finding suggests the expertise manipulation affected specifically the placement of critical objects, but not distractor objects.

Study 3 implies that the relation between perceived expertise and spatial proximity is bidirectional: A target’s expertise is not only inferred from proximity to domain-specific objects; rather if expertise is already assumed, domain-specific objects are expected to be located proximally to the target.

The findings of Study 3 illustrate two key points. First, they support the fundamental notion of a relationship between the attribution of expertise to a person and their spatial proximity to relevant objects. Second, this relationship is general in nature, being bidirectional in its effects. Additionally, the findings provide insight into the mechanisms underlying the relationship between spatial proximity and expertise. If object placement were based solely on deliberate evaluation, there would be no reason, within our experimental framework, to position the objects near the experts. Expert status alone justifies the object’s presence in the environment, as it serves as a symbol of that status. Since the co-presence of expert and object in the same space (in this case, a room) already implies their relationship, further proximity is not necessary. Consider the example of a photographer: a camera naturally belongs in their environment, as both a tool and a symbol of their profession. Whether it is placed directly beside the person or a few meters away should make no difference, however. Yet, participants consistently placed such objects close to the targets, suggesting an intuitive association process—conceptually related items are grouped together.

It should be noted that these considerations were only partially tested. As there was no option to position the critical object outside the room, placing it within the room was the only way for participants to express the expert-object relationship. Consequently, the results should be interpreted with caution, and further research is required.

Another potential limitation of the study is the inability to assert with certainty that the effect was specifically driven by expertise. Although the targets’ descriptions were carefully designed to convey expertise and validated in a pre-test, perceived expertise was not directly measured during the main experiment. Moreover, it is possible that assumed liking, an impression likely related to perceived expertise, may have contributed to the observed effects.

In the following experiment, we sought to test our assumptions in a more applied context. Previous research has repeatedly confirmed inferred expertise as a central trait of persuasive communicators. Building on this, we investigated whether a communicator (the target) would be more successful in changing attitudes when positioned proximally to the object.

## 5 Study 4

Expertise as a communicator characteristic has been of recurring interest ever since persuasion evolved as a research field in social psychology (Hovland et al., 1953). Researchers from early work onward have shown that experts are regarded as knowledgeable and therefore as reliable source of information (Hovland et al., 1953; Petty et al., 1981; Wallace et al., 2020). As numerous studies on attitude change have demonstrated, this makes experts persuasive communicators (e.g., Chaiken and Maheswaran, 1994; Petty et al., 1981; Ratneshwar and Chaiken, 1991). For recipients to attribute expertise to the communicator, researchers typically employed cues, such as an academic title or affiliation with an academic institution (but see Bohner et al., 2002; Heesacker et al., 1983; Petty et al., 1981). Here, we take a different approach and test whether proximity alone can produce a similar effect.

Building on our previous findings (Studies 1 and 2) that objects in a target’s environment, particularly in close spatial proximity, influence expertise attributions, Study 4 aims to test the influence of spatial proximity on persuasion. Specifically, we hypothesized that a communicator’s proximity to an attitude object would enhance their persuasiveness, producing effects comparable to other established expertise cues. We expected this effect to be mediated by attributed expertise.

Observers may infer a person’s liking for an object based on their spatial proximity to it (cf. Zogmaister et al., 2023). To illustrate, someone near a camera may be assumed to have both expertise and interest in photography. We expected that proximal (vs. distal) positioning would increase perceived liking, and that this perceived liking would partially account for the effect of proximity on attitudes. The corresponding preregistration is available at https://osf.io/hke58/?view_only=d42c6397b2e141d88ec4d1ec245e3f5c.

### 5.1 Method

#### 5.1.1 Participants and design

In total, the sample comprised 129 participants. One participant completed the study twice. The data of that person’s second trial were excluded from the analyses. The data of another nine participants were dropped due to their previous participation in a prior study (*n* = 8) or because they took less than a minute to participate (*n* = 1). Accordingly, a total of *n* = 119 cases (54 female, 65 male; *M*_*age*_ = 33.09, *SD*_*age*_ = 11.40) were subjected to the analyses. Participants were recruited via Prolific (see Footnote 1) and compensated with EUR 0.51. They were randomly assigned to the conditions of a 2 (distance: proximal vs. distal) × 2 (advice: in favor vs. against) between–subjects design. The power analysis for a two-way ANOVA (using G*Power 3.1; Faul et al., 2007), with distance and advice as fixed factors, returned a target sample size of 128 participants to achieve a power (1–β) of 0.80 for detecting a medium-sized effect *f* = 0.25 at a standard 0.05 alpha error probability.

#### 5.1.2 Procedure and dependent variables

Participants learned they would be participating in a study ostensibly on person perception in situations where information is limited. Following a brief overview of the general proceedings, participants were presented with an alleged screenshot of a website. The image showed a target and an object (a vacuum cleaner), and no additional information about the target was provided. Depending on the assigned condition, the spatial *distance* between the target and object was either proximal, depicting them right next to each other, or distal, depicting both at opposite edges of the image. A short message below the picture displayed the target’s *advice*, either for or against the object (“I [do not] recommend the vacuum cleaner XT-100.”). Further below, two blurred text lines were shown to give the impression of a comparison website with detailed test results (see Supplementary material). The picture and message were displayed for at least 5 s before participants could proceed.

Next, eight questions appeared sequentially below the blurred text. The initial four questions assessed participants evaluations of the object, that is, their *attitude* toward it (e.g., “How do you like the vacuum cleaner?,” 1 = *not at all* to 7 = *very much*). Participants were then asked to rate the target’s *expertise* (e.g., “How familiar is the woman with vacuum cleaners?,” 1 = *not at all* to 7 = *very much*) and the target’s *liking* for objects like the depicted, (e.g., “In your opinion, how much does the woman like to deal with vacuum cleaners?,” 1 = *very reluctant* to 7 = *very fond*), using two items for each construct. The expertise and liking questions were presented in an individually randomized order. Finally, we thanked and debriefed participants.

### 5.2 Results and discussion

We first computed a single attitude index by averaging the four items measuring participants’ attitudes (Cronbach’s α = 0.84). Similarly, we calculated expertise (Spearman-Brown ρ = 0.79) and liking indices (Spearman-Brown ρ = 0.63), both comprising two items. Table 2 summarizes participants’ averaged attitudes toward the attitude object as well as their perceptions of the communicator’s expertise and liking for objects similar to the attitude object.

**TABLE 2:** Means and standard deviations for attitude, expertise, and liking.

Variable	Advice in favor				Advice against			
	Proximal distance		Distal distance		Proximal distance		Distal distance	
	*M*	*SD*	*M*	*SD*	*M*	*SD*	*M*	*SD*
Attitude	3.48	1.14	3.04	1.00	2.57	1.04	2.93	0.89
Expertise	4.10	1.55	3.90	0.89	4.43	1.47	3.94	1.01
Liking	4.17	1.40	3.76	1.18	4.19	1.26	3.69	1.17

*N* = 119. The table displays participants’ mean attitudes toward the object as well as their mean ratings of the communicator’s perceived expertise regarding the object and the communicator’s perceived liking of the object. Higher values indicate more positive attitudes as well as higher perceived expertise and liking, respectively. All item scales ranged from 1 to 7.

#### 5.2.1 Manipulation check

Next, we checked whether participants ascribed more expertise to the communicator who was positioned proximally to the object than to one who was positioned distally. We computed a univariate ANOVA on expertise with distance (proximal vs. distal) and advice (in favor vs. against) as between–subjects factors. Unexpectedly, the analysis revealed no significant main effect of distance, *F*(1, 115) = 2.12, *p* = 0.148, η^2^ = 0.02. Moreover, no effects emerged for advice, *F*(1, 115) = 0.621, *p* = 0.432, η^2^ = 0.01, or the distance × advice interaction, *F*(1, 115) = 0.353, *p* = 0.554, η^2^ = 0. Accordingly, participants’ expertise ratings for the target were independent of the distance between the communicator and the attitude object, the valence of the message, and their interaction.

#### 5.2.2 Attitudes

**FIGURE 1:**
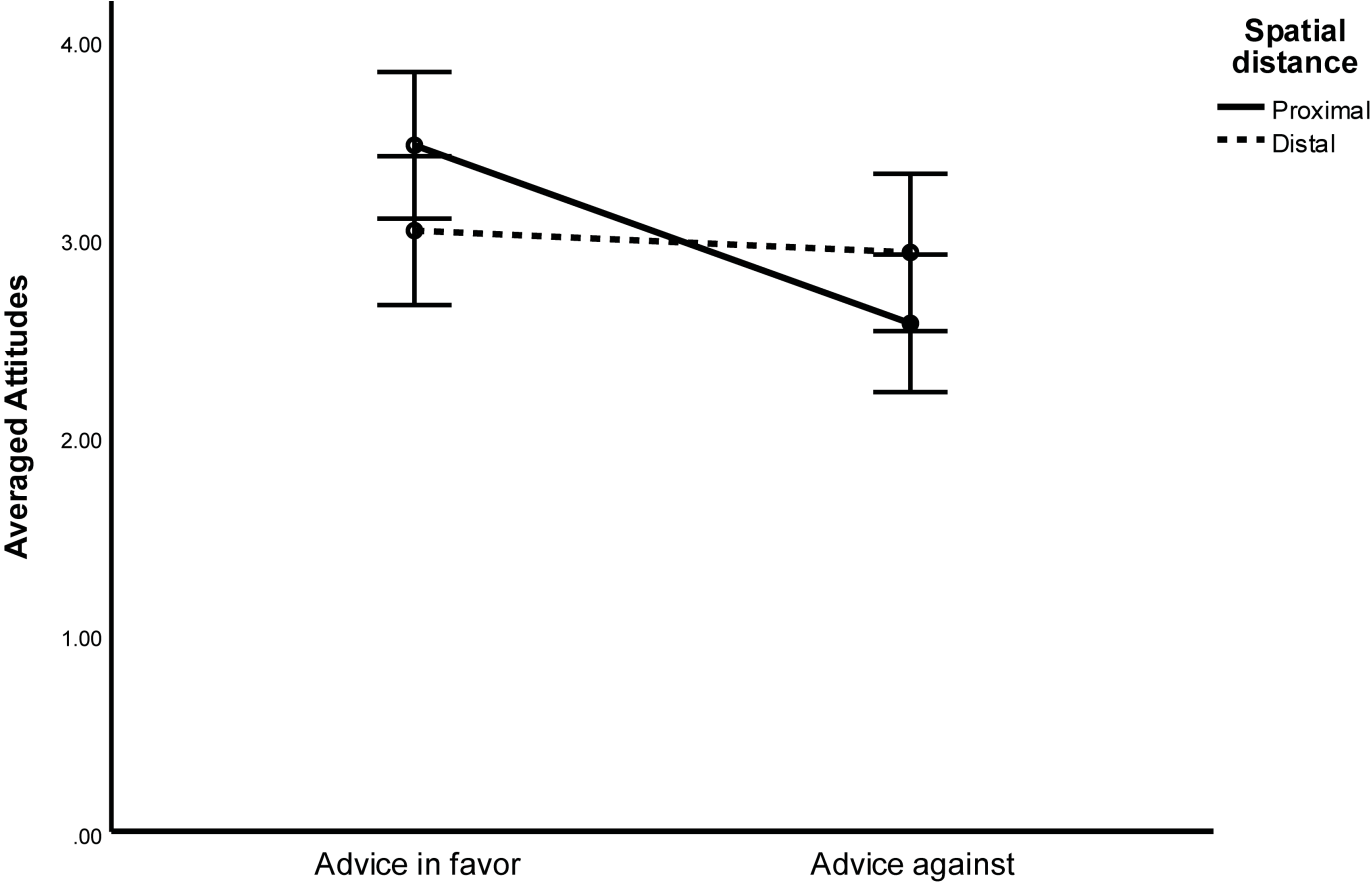
*N = 119*. The figure depicts participants’ attitudes toward the attitude object depending on the spatial proximity between the communicator and the attitude object (proximal vs. distal) and the communicators’ advice (in favor vs. against). Attitudes were measured using a total of four items; higher values represent more positive attitudes. Error bars show 95% confidence intervals.

To test our prediction regarding the influence of spatial proximity on attitude, we conducted a univariate ANOVA on attitude with distance (proximal vs. distal) and advice (in favor vs. against) as between–subjects factors. As hypothesized, the distance × advice interaction reached statistical significance, *F*(1, 115) = 4.39, *p* = 0.038, η^2^ = 0.04 (see Figure 1). Moreover, we found no main effect for distance, *F*(1, 115) = 0.05, *p* = 0.848, but for advice, *F*(1, 115) = 7.18, *p* = 0.008, η^2^ = 0.06. The latter effect indicates that the persuasive advice generally worked as intended: when the advice was in favor of the object, it led to a more positive evaluation. More importantly, however, the effect is qualified by distance: when the communicator is closer to the object, the advice becomes even more effective (see Figure 1 and Table 2).

#### 5.2.3 Mediation and correlational analyses

While we suggested that the effect of spatial proximity on attitude might be mediated by perceived expertise and liking, our data did not provide clear evidence for this mechanism. In addition to the manipulation check, which showed no significant effect of spatial proximity on perceived expertise, we also conducted an ANOVA on liking, *F*(1, 115) = 3.82, *p* = 0.053 (see Supplementary material for further details).

Due to the marginal effect of spatial distance on liking, we conducted an exploratory moderated mediation analysis (PROCESS Model 8; Hayes, 2018) with argument valence as moderator of the a- and c’-path, thereby going beyond our preregistration. The index of the moderated mediation was not significant *b* = −0.02, 95% percentile BootCI [−0.25, 0.21], indicating that the data provided no evidence for a moderated mediation through liking. We did, however, find a significant moderation of the direct effect, *b* = 0.81, *p* = 0.028, 95% percentile BootCI [0.09; 1.53]. This finding shows that the effect of spatial distance on attitude differed across levels of advice, although simple slopes were not significant within either condition (both confidence intervals included zero; see Supplementary material for further details).

Given that the a-path from spatial distance to expertise (and liking) was not significant (Hayes, 2018), we did not proceed with mediation analysis. Instead, we explored the relationships between perceived expertise, liking, and participants’ attitudes separately by argument valence (see Table 3). The correlations between perceived expertise and attitude, as well as between ascribed liking and attitude, were significant only when the communicator’s message was in favor of the object, but not when it was against it. Thus, favorable attitudes toward the object were associated with higher attributions of expertise and liking when the communicators advocated for the object.

**TABLE 3:** Correlations between ascribed expertise, liking, and attitude, separately by argument valence.

Variable	Advice in favor^a^			Advice against^b^		
	1	2	3	1	2	3
Attitude	—	0.50** [0.28, 0.67]	0.39** [0.14, 0.58]	—	0.12 [−0.13, 0.37]	0.15 [−0.12, 0.39]
Expertise		—	0.71** [0.55, 0.82]		—	0.61** [0.42, 0.75]
Liking			—			—

*^a^n* = *59*,

*^b^n* = *60.* The table shows Pearson’s *r* coefficients for the associations of ascribed expertise and liking with participants’ attitudes, reported separately by argument valence.

**p < 0.01.

Study 4 supported the hypothesis that a communicator in close spatial proximity to an attitude object is more persuasive than one positioned at a distal distance. Participants indicated more favorable attitudes when the communicator advocating in favor of the attitude object was positioned proximally (vs. distally) to it. The same pattern emerged when the communicator advocated against the attitude object. Therefore, proximity to an attitude object may function as a cue equivalent to other expertise cues used in persuasion research, such as academic titles (e.g., Petty et al., 1981), by producing similar outcomes.

Unexpectedly, the manipulation check revealed no direct effect of spatial proximity on expertise. A plausible explanation for this finding might be individuals’ propensity to base their judgments on highly accessible information (for an overview, see Förster and Liberman, 2007; Higgins, 1996; Wyer and Srull, 1989). Since participants indicated their attitude toward the object immediately before assessing the communicator’s expertise, they may have relied on their attitude when judging expertise. This question order was deliberately chosen to avoid artificially enhancing the salience of the expertise cue. However, this approach comes with a trade-off: by measuring expertise only after attitude, the effect of spatial proximity, manipulated at the beginning of the experiment, may have dissipated (Fayant et al., 2017) or been overridden by the salience of participants’ attitudes. As a result, our design limited the possibility of detecting an effect of the independent variable and establishing causal mediation. Alternatively, the preceding attitude judgment may have introduced a competing source of information that reduced the influence of spatial proximity on expertise judgments.

Although the robust correlation between expertise and liking suggests a close connection between the two constructs, the relatively small sample sizes warrant caution in interpreting this finding. Surprisingly, the link between attitudes and expertise or liking only emerged when the communicator advocated for the attitude object. One possible explanation why no such association was observed when the communicator argued against the object could again be that participants relied on their own attitudes when evaluating the communicator’s expertise (e.g., “I didn’t like the product. This may be because the communicator lacked expertise on the object and therefore failed to persuade me.”). Another possibility is that the advertising-like format may have been confusing, particularly in the condition where the communicator argued against the object. The expectation of persuasive intent typically implies promotion, and a communicator dissuading from a product in such a format may have appeared unusual or less credible to participants.

Although the predicted effect of spatial proximity on persuasiveness was observed, the failed manipulation check raises the possibility that this effect was driven by processes other than those initially hypothesized. Still, the absence of a direct effect of spatial proximity on expertise may be a methodological artifact rather than evidence that the proposed mechanism does not occur. In fact, the observed persuasion effect suggests that spatial cues may have shaped expertise judgments in line with our assumptions—even if this influence could no longer be reliably captured by our measure.

The significant interaction indicates that the association between spatial distance and attitudes differed as a function of advice, with simple slopes suggesting opposite directional patterns, although neither reached statistical significance.

Study 4 confirmed the relevance of spatial proximity in a real-world persuasion context. Attitudes varied as a function of the interaction between proximity and message valence: Communicators were more persuasive when positioned in proximity to the attitude object, regardless of message position. This pattern is consistent with an account based on perceived expertise, although the failed manipulation check prevents strong conclusions regarding the underlying mechanism.

## 6 General discussion

To identify sources of reliable information or competent advice, individuals often rely on various cues—including, as the present research suggests, a person’s spatial proximity to a relevant object. In this research, we investigated the hypothesis that individuals use spatial proximity to deduce another person’s expertise related to an object.

Across four experiments, we found converging evidence that spatial proximity plays a causal role in how expertise is inferred. Study 1 showed that individuals infer domain-specific expertise from the presence (vs. absence) of relevant objects near a person. Study 2 demonstrated that targets positioned in proximity (vs. at a distance) to an object are attributed greater expertise in the related domain. Study 3 complemented this finding by showing the reverse inference: individuals deduce a person’s spatial proximity to an object based on that person’s expertise. Finally, Study 4 revealed that a communicator’s proximity to an attitude object can increase persuasion effectiveness.

The current investigation corroborates evidence that spatial proximity can serve as an informative cue. It allows individuals to infer relationships between entities and attribute relevant characteristics based on spatial proximity (Chae et al., 2013; cf. Hall, 1969, 1973; Wellens and Goldberg, 1978). Extending prior findings to the domain of interpersonal judgment, our findings show that individuals rely on perceived spatial proximity to infer a person’s expertise: the more proximal the person is to an object, the more expertise is ascribed to them.

Furthermore, our findings contribute to research on object-based person perception by introducing expertise as an attribute inferred from objects in spatial proximity (Gosling et al., 2002; Naumann et al., 2009; Oh et al., 2020; Rentfrow and Gosling, 2006). Prior work (e.g., Gosling et al., 2002) has shown that observers form impressions of traits based on objects located in personal environments such as offices or bedrooms, contexts in which ownership of the objects is likely assumed. Consistent with this line of research, in our study, participants attributed expertise to targets positioned proximally to domain-relevant objects. Importantly, across both prior and current studies, the *specific* spatial arrangement of objects appears less critical, as the mere presence of an object within relevant space is sufficient to guide trait or skill judgments. This categorical perspective is supported by Study 1, where the presence of an object, rather than its absence, led to higher expertise attributions. However, the present work complements this categorical perspective by highlighting a more dimensional view, in which spatial proximity itself can act as a meaningful cue and expertise attributions vary as a function of how close a target is positioned to an object. Specifically, in Study 2, participants ascribed more expertise to targets positioned proximally (vs. distally) to domain-relevant objects; in Study 3, they placed such objects closer to targets with matching expertise. The overall effect for mere presence in Study 1 was considerably larger than the effect for relative spatial proximity observed in Study 2. This difference likely reflects the fact that Study 2 contrasted a very small proximal distance (target and object positioned directly adjacent) with a slightly larger, though still relatively small, distance (target and object positioned at opposite image borders). Notably, the effect emerged despite these objectively minimal spatial variations, consistent with the idea that individuals rely on objects (that are present) in another person’s spatial proximity when attributing expertise.

Taken together, the results of the present investigation suggest that perceived expertise may not only depend on the presence of an object, but also on its spatial proximity to the person. Finally, our results align with well-established findings in persuasion research: communicators who are perceived as experts, here, by virtue of being positioned proximally to a relevant object, are more persuasive than those lacking such perceived expertise (Chaiken and Maheswaran, 1994; Kruglanski and Thompson, 1999; Petty et al., 1981).

The present findings suggest that spatial proximity to an object influences expertise attributions. We propose that this attribution follows a two-step process. First, individuals categorize a person based on salient contextual cues, such as a domain-relevant object (Fiske and Neuberg, 1990). Second, they interpret the spatial proximity between the person and the object as indicative of their relationship. Spatial proximity suggests a close relationship and familiarity (Chae et al., 2013; Hall, 1969, 1973), which may prompt the attribution of expertise in that domain to the person. This reasoning partly builds on Chae et al. (2013), who drew on Michotte (1963). The authors argued that people generalize learned mechanical cause-effect patterns, like the assumption that closely positioned entities are causally related, to make more abstract judgments, such as evaluating the effectiveness of an insecticide. Although our results are consistent with the general idea, our studies do not yet allow for firm conclusions about the underlying psychological mechanisms.

The present findings may also have implications for applied contexts, particularly in settings where more overt or effortful indicators of expertise are unavailable. We emphasized earlier that conventional cues, such as academic titles, occupational attire, or formal credentials, often require domain-specific or culture-specific knowledge to be correctly interpreted and are confined to certain domains. Our findings suggest, by contrast, that perceived expertise can be shaped by spatial proximity, a cue that may operate independently of culture-specific knowledge and be applicable across a broader range of contexts.

This insight could be applied in various contexts, for instance, in designing advertising for a specific product, in efforts to persuade an audience to adopt a health-promoting behavior, or to underscore one’s expertise in a job interview—by placing the advertising figure, a communicator, or oneself in proximal spatial relation to relevant objects, such as a camera, a basket of healthy foods, or a psychology textbook.

## 7 Limitations and future research

The present research provides empirical evidence identifying spatial proximity as a relevant cue for attributing expertise. While our studies offer novel insights into how people use spatial cues to make social judgments, larger sample sizes would allow for more robust interpretation of the results. Beyond these contributions, our findings raise important questions that warrant further investigation.

The most significant issue concerns the psychological mechanisms underlying the spatial proximity effect. Our primary objective was to test whether the hypothesized (basic) spatial proximity effect exists. Establishing the existence of this effect represents a necessary first step, providing a foundation for future work to examine the processes by which spatial proximity informs expertise attribution. While our findings are consistent with the proposed two-step account, comprising initial categorization followed by a proximity-based inference of a relationship, the empirical support for this process remains preliminary. Future studies should examine the first step of our proposed process, the activation of a relevant category. For example, it remains an open question under which conditions a category becomes salient, including what types of objects are sufficient to trigger this categorization. A further open question concerns whether spatial proximity effects are restricted to more intuitive forms of judgments or also extend to more deliberate, effortful evaluations (Kruglanski and Gigerenzer, 2011). Finally, it remains unclear how broadly inferred expertise is applied, that is, whether it extends beyond the presented object to non-presented objects from the same category or is restricted to the specific object shown (cf. Glaser et al., 2015; Linne et al., 2020). Initial exploratory results suggest that expertise attributed to, for instance, dogs does not transfer to cats (Study 1). Beyond this, it is important to consider boundary conditions of the spatial proximity effect. These include contextual information or prior knowledge about the target that may render the attribution of expertise in a given domain unlikely *despite* the target’s close spatial proximity to a relevant object. For instance, a child standing near a fire would not be perceived as an expert firefighter, as most individuals know that children are not typically firefighters. Likewise, in domains involving hazardous materials, maintaining distance from an object may itself signal domain expertise (when observers recognize the associated risks). In addition, observers’ own expertise may shape their judgments. For example, a person standing next to a brush and a freshly painted wall might be perceived as a painter; however, the object in question is a paste brush typically used for applying wallpaper adhesive rather than for painting walls—a detail that may go unnoticed by lay observers.

Building on prior work (e.g., Gosling et al., 2002; Naumann et al., 2009), future research may explore whether the role of spatial proximity extends beyond expertise. If spatial distance serves as a general cue for perceived relationships between people and objects, similar effects may arise for other inferences. It seems plausible that proximity to an object not only signals expertise in the corresponding domain but also evokes judgments about traits associated with that object. For example, a person positioned near a fitness device might be perceived as healthier than someone located farther away.

Another promising direction for future research concerns the role of spatial proximity in persuasion. Study 4 demonstrated that proximity to an attitude object can influence persuasion, even when only minimal evaluative information is presented. Building on this, future studies might examine whether and how spatial proximity interacts with more elaborate judgment-relevant arguments or information. Drawing on dual-process models of persuasion, such as the elaboration likelihood model (ELM; Petty and Cacioppo, 1986) and the heuristic-systematic model (HSM; Chaiken et al., 1989), a general assumption can be derived: when recipients are sufficiently motivated and able to engage in effortful processing, peripheral or heuristic cues (e.g., perceived expertise) can influence attitudes by serving as additional arguments or bias the processing of information (Petty and Wegener, 1998; Tormala et al., 2007). This influence is particularly pronounced when arguments are ambiguous or open to interpretation (Chaiken and Maheswaran, 1994; Erb et al., 2007). Accordingly, participants may form more favorable attitudes toward an attitude object either because they interpret the communicator’s spatial proximity as a sign of expertise, thereby treating this perceived expertise as an additional argument, or because they are more inclined to process the message favorably when they believe it originates from an expert. Prior research on communicator expertise and cue-based processing provides a strong foundation for further investigating how spatial proximity operates in more elaborate persuasive contexts (Bohner et al., 2002; Chaiken and Maheswaran, 1994; Erb et al., 2007). Investigating the role of spatial proximity in more information-rich contexts is particularly warranted, given that the materials used in Study 4 were relatively minimalistic. In applied settings such as marketing, however, persuasive messages typically involve a broader range of informational cues.

## 8 Conclusion

The present research demonstrates a relationship between spatial proximity and the attribution of expertise. Individuals tend to infer a person’s expertise from their spatial proximity to a relevant object, such that being proximal gives rise to perceptions of expertise. For example, a reporter located within a region may be seen as more expert on its political affairs than someone covering it remotely; similarly, a person standing near a car may be perceived as having more expertise in cars than one situated farther away. Thus, formal credentials or attire are not always necessary to convey expertise, spatial proximity to a relevant object may be enough.

## Data Availability

The datasets presented in this study can be found in online repositories. The names of the repository/repositories and accession number(s) can be found below: https://doi.org/10.17605/OSF.IO/EGZ2F.

## Footnotes

1
https://www.prolific.com
