# Supplementary material for: Close is clever! Spatial proximity shapes expertise attribution
Source: Front Psychol. 2026 Aug 24;17:1763841. doi: 10.3389/fpsyg.2026.1763841 (PMC13547489; doi:10.3389/fpsyg.2026.1763841)
Supplement: Supplementary file 1 [file Supplementary_file_1.pdf]

Table 1

Deviations from Preregistrations

Study	Deviation
2	The preregistration also describes an additional experiment that is not included in the present manuscript (referred to as "Study 1" in the preregistration). That experiment is not included because the initial research phase had a broader focus on spatial proximity effects, whereas the present manuscript focuses specifically on target–object configurations.
3	The preregistration specified an additional variable ('motivation' as control variable) that was not assessed in the present study; consequently, no analyses involving this variable are reported.
4	The preregistration specified an independent *t*-test to examine the effect of spatial proximity on attributions of expertise. Given the factorial design, we instead conducted a two-way ANOVA including spatial distance (proximal vs. distal) and advice (in favor vs. against) as factors.

Study 1

The Supplementary Material presents example pictures of the stimulus material (Figure 1) and additional results supporting the main findings of Study 1. They include graphical representations of expertise ratings across all trials, displayed separately for the object-present and object-absent conditions (Figure 2), detailed results of independent-samples *t*-tests conducted separately for each trial and object condition (Table 2), and means and standard deviations for all three expertise domains of each trial (Table 3).

Figure 1

Example Stimuli Used in Study 1 Depicting the two Levels of the Object Manipulation (Present vs. Absent)

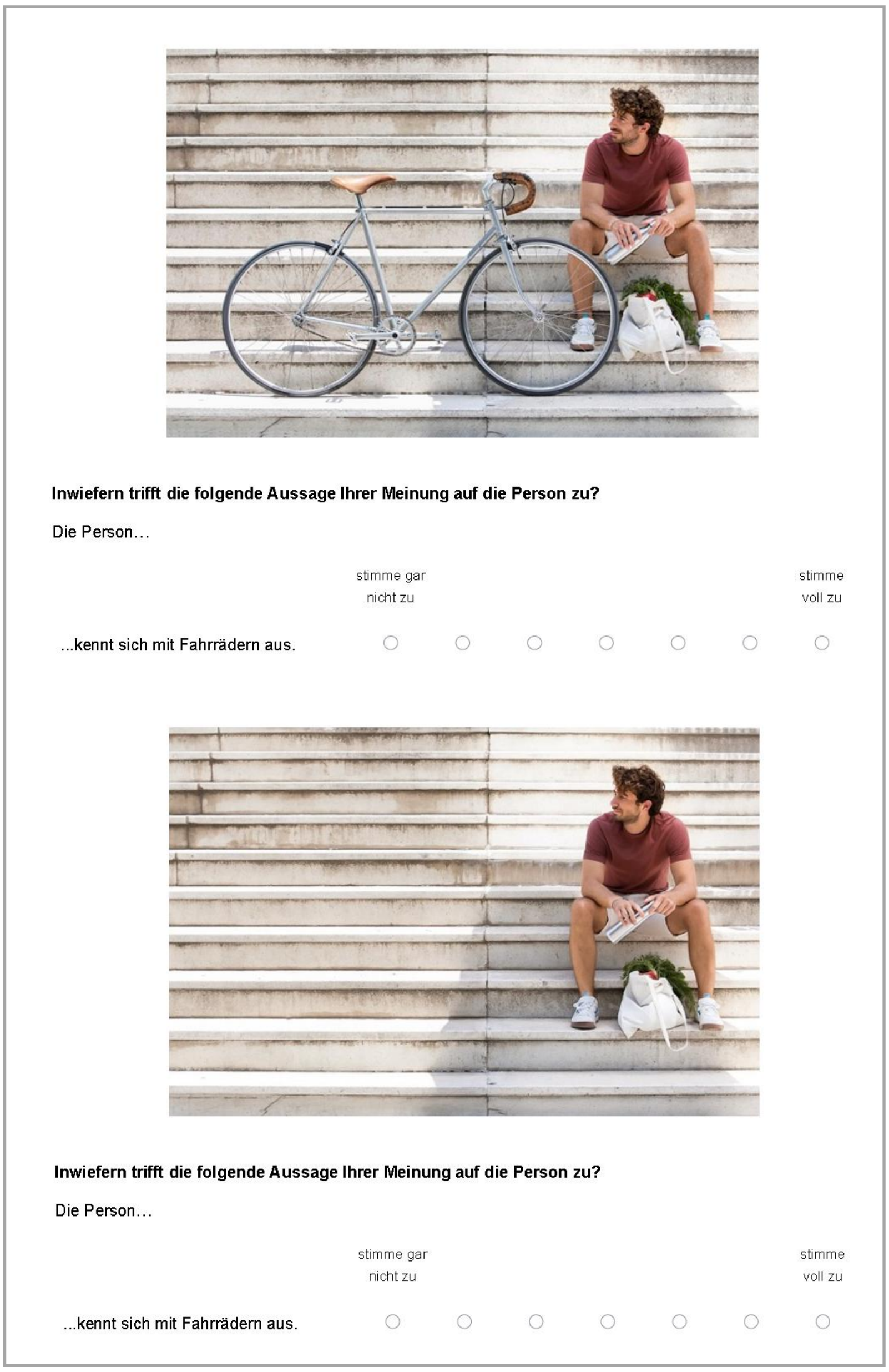

Note. The figure shows the two variants of one of the seven targets. All images are license-free and were modified so that the targets were presented either with an object (e.g., a bicycle, a dog, a canoe) or alone. Below the pictures, six different statements about the respective target were displayed sequentially.

Figure 2

Averaged Expertise Ratings Separate for Present and Absent Object Across Trials

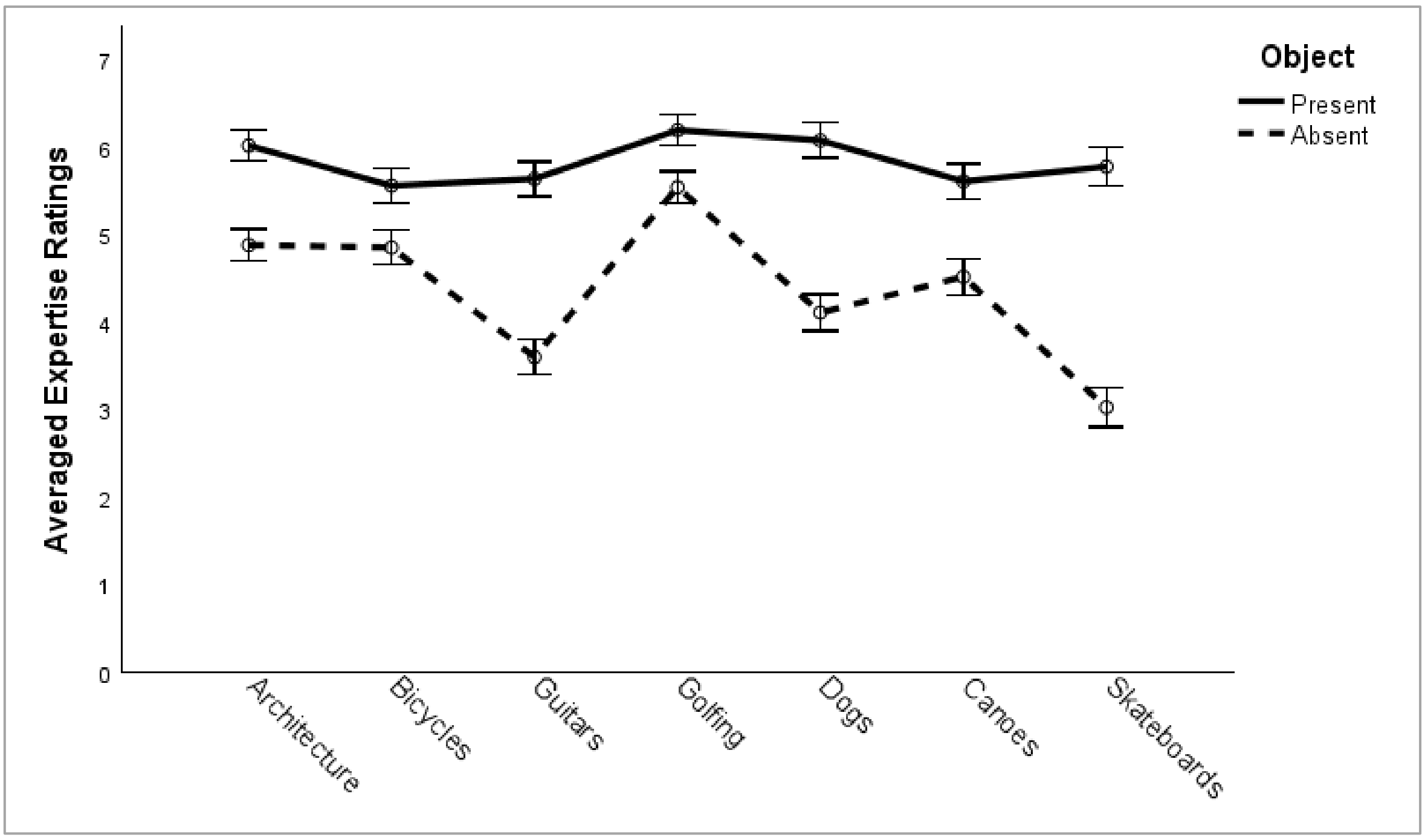

Note. $N = 269$. The figure displays mean expertise ratings for targets presented with (present) and without (absent) objects, shown separately for each of the seven trials. Error bars show 95 % confidence intervals. Ratings were provided on a 7-point scale ranging from 1 = *completely disagree* to 7 = *completely agree*.

Table 2

Averaged Expertise Ratings Separate for Present and Absent Objects Across Trials

Trial	Object		$t(267)$	$p_{one\text{-}tailed}$	*Cohen's d*
	Present	Absent			
Architecture	6.01 (1.02)	4.88 (1.08)	8.84	< .001	1.08
Bicycles	5.55 (1.01)	4.85 (1.26)	5.08	< .001	0.62
Guitars	5.64 (0.98)	3.60 (1.35)	14.12	< .001	1.73
Golfing	6.19 (0.80)	5.54 (1.28)	4.99	< .001	0.61
Dogs	6.07 (0.81)	4.11 (1.50)	13.32	< .001	1.64
Canoes	5.61 (1.04)	4.52 (1.36)	7.37	< .001	0.90
Skateboards	5.77 (1.18)	3.02 (1.42)	17.22	< .001	2.11

Note. $N = 269$. Levene's test indicated equal variances for the *architecture* and *bicycles* trials; therefore, pooled-variance *t*-tests were used for these comparisons. Welch's correction was applied for all remaining domains.

The following results are from exploratory analyses on the proximity–expertise effect across common and special expertise domains. For the common expertise (bicycles), ratings were higher when the bicycle was present (*M* = 5.55, *SD* = 1.01) than absent (*M* = 4.85, *SD* = 1.26), mean difference = 0.71, *SE* = 0.14, $p < .001$, 95% CI [0.43, 0.98]. For the special expertise (architecture), ratings were higher when the construction plan was present (*M* = 6.01, *SD* = 1.02) than absent (*M* = 4.88, *SD* = 1.08), mean difference = 1.14, *SE* = 0.13, $p < .001$, 95% CI [0.88, 1.39].

The following analyses provide a more detailed examination of the domain type × object interaction reported in the main text, including linear and quadratic trends, descriptive statistics, and pairwise comparisons. Table 3 shows means and standard deviations for all three expertise domains across targets, separately for each object condition. Table 4 presents the results of the mixed ANOVA on expertise, including main effects of object and domain type, as well as the domain type × object and trials × object interactions.
Contrast analyses showed a significant linear trend across domain types, $F(1, 267) = 1057.84$, $p < .001$, $\eta^2_p = .80$, and a significant quadratic trend, $F(1, 267) = 375.22$, $p < .001$, $\eta^2_p = .58$. A significant domain type × object interaction emerged for both the linear, $F(1, 267) = 277.68$, $p < .001$, $\eta^2_p = .51$, and the quadratic trend, $F(1, 267) = 153.60$, $p < .001$, $\eta^2_p = .37$.

When objects were present, the primary decline in expertise ratings occurred between depicted (*M* = 5.84, SE = 0.06) and to related domains (*M* = 3.65, *SE* = 0.06); ratings for unrelated domains (*M* = 3.50, *SE* = 0.06) were only marginally lower. A similar but weaker pattern emerged when objects were absent: ratings dropped from depicted[1] (*M* = 4.36, *SE* = 0.06) to related domains (*M* = 3.76, *SE* = 0.06), with unrelated domains (*M* = 3.61, *SE* = 0.06) again only slightly lower. Pairwise comparisons of estimated marginal means, averaged across the seven trials, indicated that expertise ratings decreased from depicted to related and unrelated domain types (depicted > related = unrelated), particularly in the object-present condition (Table 5). Unexpectedly, an ordering of domain types also emerged in the object-absent condition.

[1] The designation may be misleading. In the “absent” condition, the critical object (e.g., the bicycle or the construction plan) was of course not visible.

Table 3

Mean Expertise Ratings for Present and Absent Objects by Domain Type Across Trials

Trial	Domain type	Object	
		Present	Absent
Architecture	Architecture[a]	6.01 (1.02)	4.88 (1.08)
	Urban planning[b]	5.30 (1.22)	4.60 (1.28)
	Marine animals[c]	2.48 (1.06)	2.73 (1.09)
Bicycles	Bicycles[a]	5.55 (1.01)	4.85 (1.26)
	Motor scooters[b]	2.99 (1.05)	3.75 (1.27)
	History[c]	3.96 (0.99)	4.11 (1.26)
Guitars	Guitars[a]	5.64 (0.98)	3.60 (1.35)
	Pianos[b]	3.42 (1.10)	3.88 (1.29)
	Healthy nutrition[c]	4.91 (0.95)	5.21 (1.17)
Golfing	Golfing[a]	6.19 (0.80)	5.54 (1.28)
	Bowling[b]	2.83 (1.20)	3.48 (1.31)
	Dinosaurs[c]	2.61 (1.22)	2.74 (1.32)
Dogs	Dogs[a]	6.07 (0.81)	4.11 (1.50)
	Cats[b]	3.16 (1.15)	3.49 (1.29)
	Gardening[c]	3.91 (1.15)	3.97 (1.49)
Canoes	Canoes[a]	5.61 (1.04)	4.52 (1.36)
	Sailboats[b]	3.99 (1.24)	3.97 (1.32)
	Stocks[c]	3.31 (1.26)	3.58 (1.24)
Skateboards	Skateboards[a]	5.77 (1.18)	3.02 (1.42)
	Skates[b]	3.85 (1.25)	3.14 (1.33)
	Home improvement[c]	3.33 (1.21)	2.90 (1.30)

Note. $N = 269$. Values represent mean expertise ratings (M) and standard deviations (SD) for each domain type, separately for the object-present and object-absent conditions across all seven trials. Ratings were made on scales from 1 = *completely disagree* to 7 = *completely agree*.
[a] Domain depicted by the object when present.
[b] Domain is conceptually associated with trial domain.
[c] Domain is conceptually independent of trial domain.

Table 4

Results of a Mixed ANOVA on Expertise

Variable	Type III Sum of Squares	*df*	Mean Square	*F*	*p*	η^2_p
(Intercept)	95748.42	1	95748.42	13435.65	< .001	.98
Object	250.02	1	250.02	35.08	< .001	.12
Domain type	2726.89	2	1363.45	798.09	< .001	.75
Domain type × Object	787.45	2	393.72	230.47	< .001	.46
Trials	331.51	5	55.25	40.55	< .001	.13
Trials × Object	254.42	6	42.40	31.12	< .001	.10

Note. $N = 269$. The table shows the results of a mixed ANOVA on expertise with object (present vs. non-present) as between–subjects factor and domain type (represented vs. related vs. unrelated) and trials (Targets 1 to 7) as within–subjects variables.

Table 5

Pairwise comparisons of Estimated Marginal Means for Expertise Ratings Across Domain Types Within Each Object Condition

Object	(I) Domain type	(II) Domain type	Mean Difference	*SE*	*p*	95 % CI	
						LL	UL
Present	Represented	Related	2.19	.06	< .001	2.04	2.34
		Unrelated	2.33	.06	< .001	2.17	2.49
	Related	Represented	–2.19	.06	< .001	–2.34	–2.04
		Unrelated	0.14	.05	.008	0.03	0.26
	Unrelated	Represented	–2.33	.07	<. 001	–2.49	–2.17
		Related	–0.14	.05	.008	–2.34	–2.04
Absent	Represented	Related	0.60	.07	< .001	0.45	0.76
		Unrelated	0.75	.07	< .001	0.59	0.92
	Related	Represented	–0.60	.07	< .001	–2.34	–2.04
		Unrelated	0.15	.05	.006	0.04	0.27
	Unrelated	Represented	–0.75	.07	< .001	–2.34	–2.04
		Related	–0.15	.05	.006	–2.34	–2.04

Note. $N = 269$. Values represent pairwise comparisons of estimated marginal means. Positive mean differences indicate higher expertise ratings for the domain listed in column (I) relative to column (II). Object condition refers to whether the object was present or absent. Expertise ratings were assessed on a scale from 1 to 7. Represented domains were architecture, bicycles, guitars, golfing, dogs, canoes, and skateboards. Related domains belonged to the same broader category as the represented domains and included urban planning, motor scooters, pianos, bowling, cats, sailboats, and skates. Unrelated domains had no conceptual connection to the represented domains and included marine animals, history, healthy nutrition, dinosaurs, gardening, stocks, and home improvement.

Study 2

Before the actual experiment started, we presented participants with the following introduction to encourage them to follow their intuition when they assess the targets:

While looking at the pictures, you may get the feeling that you do not have enough information about the persons depicted. However, the findings of previous research imply that individuals do not need many cues to form an impression of another person. Individuals also seem to take the external appearance of the person as well as the surrounding of the person into their judgment. The way in which the person and their surroundings are portrayed, whether in a photograph, a historical painting, or a cartoon, does not seem to play a role—individuals are always able to extract information, such as, characteristics or even interests, about depicted person.

Each picture will be displayed for 5 seconds. Subsequently, questions pertaining to the depicted person will appear one after another underneath the picture. When answering these questions, you are welcome to follow your intuition or spontaneous sensations. There are no right or wrong answers to these questions. It is your personal assessment that is important to us. When you have chosen one of the answer options, the next question will appear automatically, or you will be redirected automatically to the next page. In order that you can become familiar with the proceeding, the investigation starts on the following page with a short trial run, which will not be included in the analysis.

Figure 3

Example Stimuli From Study 2 Illustrating the Proximal and Distal Levels of Spatial Proximity

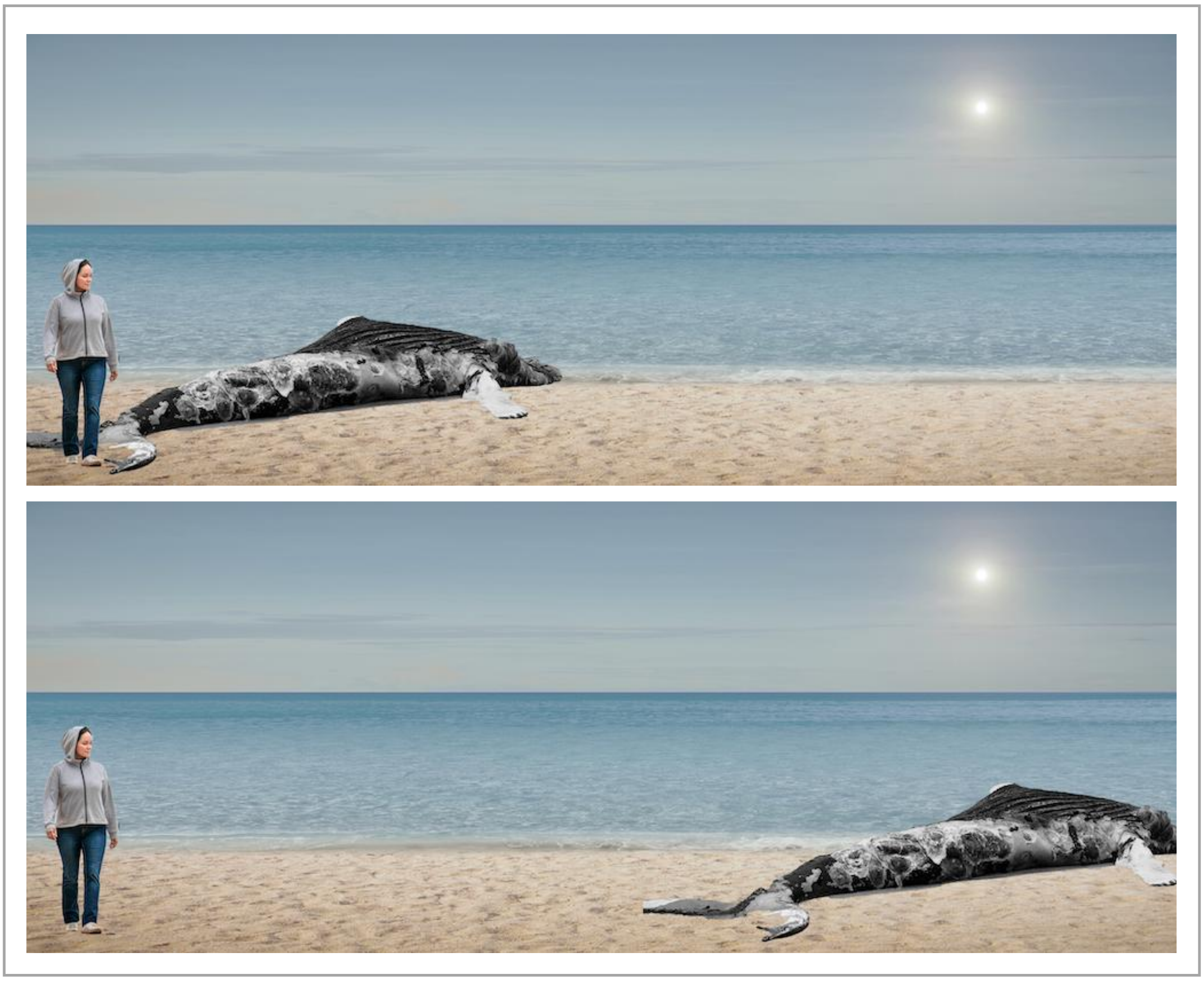

Note. The picture shows two example stimuli from the four critical trials, with each target presented in either proximal or distal spatial proximity to the respective object (e.g., a whale carcass, a car, a robot). Below the pictures, ten different questions about the depicted target were displayed one after the other (e.g., “How do you rate the woman’s expertise in marine animals?”, 1 = *no expertise* to 7 = *high expertise*).

As a post-hoc analysis, we computed an independent *t*-test for each trial to compare the expertise ratings for each target person as a function of the spatial distance between the target person and the object. The results are listed in Table 6.

Table 6

Averaged Expertise Ratings Separate for Proximal and Distal Distances Across Critical Trials

Critical trial	Distance		$t(96)$	$p_{one\text{-}tailed}$	*Cohen's d*
	Proximal	Distal			
Car	5.33 (1.17)	4.76 (1.15)	2.47	.008	-.49
Robot	5.65 (1.51)	5.38 (1.39)	0.90	.186	-.19
Nuclear waste	5.37 (1.50)	4.83 (1.48)	1.80	.037	-.36
Whale carcass	4.76 (1.78)	4.50 (1.40)	0.82	.209	-.16

Note. $N = 98$. The table lists expertise ratings participants attributed on average to the targets of the four critical trials. The employed scales measuring expertise ranged from 1 = *low expertise* to 7 = *high expertise*. Standard deviations are in parentheses.

Table 7

Averaged Similarity Ratings Separate for Proximal and Distal Distances Across Critical Trials

Critical trial	Distance	
	Proximal	Distal
Car	2.41 (1.27)	2.51 (1.16)
Robot	3.35 (1.46)	2.81 (1.31)
Nuclear waste	3.20 (1.51)	3.30 (1.30)
Whale carcass	3.06 (1.63)	3.15 (1.57)

Note. $N = 98$. The table displays participant's perceived similarity toward targets. Similarity was assessed using a single item, ranging from 1 = *not at all similar* to 7 = *very similar*. Standard deviations are in parentheses.

Table 8

Results of Independent-Sample t-Tests for Distractor Items Utilized in Study 2

Item	t	Bonferroni corrected p-value
Car		
How helpful do you think this man is?	–1.57	1
How independent do you consider the man?	0.71	1
In your opinion, how responsible is the man?	–1.57	1
How interested do you think is the man in soccer?	1.07	1
How well does the man know classical music?	–1.22	1
Robot		
In your opinion, how active do you consider the man?	0.61	1
How outgoing would you consider the man?	–1.41	1
How diverse do you think the man's interests are?	0.50	1
How well does the man know tennis?	–0.95	1
How much do you think the man is interested in cooking?	–0.20	1
Nuclear waste		
How content does the woman seem to you?	0.47	1
How optimistic do you think the woman is?	–1.15	1
In your opinion, how caring do you think this woman is?	–1.09	1
How do you estimate the woman’s interest in playing board games?	–0.44	1
How well does the woman know modern literature?	–1.50	1
Whale carcass		
How adventurous does this woman seem to you?	1.97	0.54
How helpful do you consider the woman to be?	1.07	1
How connected to nature do you think the woman is?	0.18	1
In your opinion, how responsible is the woman?	0.67	1
How much do you think the woman is interested in interior design?	–1.31	1

Note. $N = 98$. The table summarizes the t-values and Bonferroni-adjusted p-values of the independent-samples t-test across all distractor items with distance (proximal vs. distal) as the independent variable. Participants had to rate each item on a seven-point Likert scale ranging from 1 representing a low manifestation of the characteristic to 7 representing a high manifestation of the respective characteristic.

Study 3

In the following, we provide the reader with all descriptions utilized in Study 3.

#1 Target person

Expertise matches the critical object (camera):

Ich würde sagen, dass mein Beruf mich ganz gut widerspiegelt. Ich bin Fotografin und habe ein eigenes Atelier. Neben dem „Alltagsgeschäft" habe ich immer wieder die Möglichkeit, meiner Kreativität freien Lauf zu lassen. Etwa wenn sich ein Paar mal ein ausgefalleneres Hochzeitsmotiv wünscht.
Ich bin ein sehr offener und kommunikativer Mensch, weswegen ich auch gern mit meinen Kunden gemeinsam überlege, in welche Richtung ihre Idee gehen soll. Aber mein Beruf hat natürlich auch eine praktische Seite, die mir große Freude bereitet. Ein Bürojob, bei dem ich den ganzen Tag vor dem Computer sitze, wäre nichts für mich. Die Fotografie macht mir einfach mehr Spaß!

I would say that my job reflects me quite well. I am a photographer and have my own studio. Besides the everyday business, I always have the opportunity to give free rein to my creativity. For example, if a couple wishes for an unusual wedding motif.

I'm a very open and communicative person, which is why I enjoy developing the direction that my customer's idea should take together with them. However, my job also has a practical side, of course, which gives me great pleasure. An office job where I sit in front of the computer all day would not be for me. Photography is simply more fun for me!

Expertise does not match the critical object (camera):

Ich würde sagen, dass mein Beruf mich ganz gut widerspiegelt. Ich bin Konditorin und habe eine eigene Konditorei. Neben dem „Alltagsgeschäft" habe ich immer wieder die Möglichkeit, meiner Kreativität freien Lauf zu lassen. Etwa wenn sich ein Paar mal eine ausgefallenere Hochzeitstorte wünscht.
Ich bin ein sehr offener und kommunikativer Mensch, weswegen ich auch gern mit meinen Kunden gemeinsam überlege, in welche Richtung ihre Idee gehen soll. Aber mein Beruf hat natürlich auch eine praktische Seite, die mir große Freude bereitet. Ein Bürojob, bei dem ich den ganzen Tag vor dem Computer sitze, wäre nichts für mich. Die Konditorei macht mir einfach mehr Spaß!

I would say that my job reflects me quite well. I am a pastry chef and have my own confectionery. Besides the everyday business, I always have the opportunity to give free rein to my creativity. For example, if a couple wishes for an unusual wedding cake.
I'm a very open and communicative person, which is why I enjoy developing the direction that my customer's idea should take together with them. But my job also has a practical side, of course, which gives me great pleasure. An office job where I sit in front of the computer all day would not be for me. The confectionery is simply more fun for me!

#2 Target person

Expertise matches the critical object (dog):

Was mich am meisten ausmacht? Vermutlich meine spontane Art und mein Optimismus. Beispielsweise habe ich mir vor einigen Jahren einen Traum erfüllt und einen Hund adoptiert. Das war in dem Moment tatsächlich eher eine

Kurzschlussreaktion, weil jemand den dringend abgeben musste. Danach habe ich dann alles gelesen, was ich in die Finger bekommen konnte und hab vieles auch einfach ausprobiert. Ich wusste, dass das schon gutgehen wird. Mittlerweile kann ich anderen in Sachen Hund mit Rat und Tat zur Seite stehen. Ansonsten würde ich mich noch als sehr kontaktfreudig beschreiben. Ich lerne gern neue Leute kennen oder verbringe Zeit mit meinen Freunden.

What makes me tick? Probably my spontaneous nature and optimism. For instance, a couple of years ago I fulfilled a dream of mine when I adopted a dog. Right at that moment, that actually was a knee-jerk reaction because someone desperately needed to hand it off. After that, I read everything I could get my hands on and tried out a lot of things. I knew that it would work out. In the meantime, I am on hand with help and advice for others when it comes to dogs. Apart from that, I would describe myself as very sociable. I like to meet new people or spend time with my friends

Expertise does not match the critical object (dog):

Was mich am meisten ausmacht? Vermutlich meine spontane, aber auch pragmatische Art. Beispielsweise habe ich mir vor einigen Jahren einen Traum erfüllt und einen Garten gepachtet. Das war in dem Moment tatsächlich eher eine Kurzschlussreaktion, weil jemand den dringend abgeben musste. Danach habe ich dann alles gelesen, was ich in die Finger bekommen konnte und hab vieles auch einfach ausprobiert. Ich wusste, dass das schon gutgehen wird. Mittlerweile kann ich anderen in Sachen Garten mit Rat und Tat zur Seite stehen. Ansonsten würde ich mich noch als sehr kontaktfreudig beschreiben. Ich lerne gern neue Leute kennen oder verbringe Zeit mit meinen Freunden.

What makes me tick? Probably my spontaneous nature and optimism. For instance, a couple of years ago I fulfilled a dream of mine when I leased a garden lot. Right at that moment, that actually was a knee-jerk reaction because someone desperately needed to hand it off. After that, I read everything I could get my hands on and tried out a lot of things. I knew that it would work out. In the meantime, I am on hand with help and advice for others when it comes to gardening. Apart from that, I would describe myself as very sociable. I like to meet new people or spend time with my friends.

#3 Target person

Expertise matches the critical object (bass guitar):

Ich spiele seit meiner Jugend Bassgitarre. Früher bin ich mit meiner Band regelmäßig bei Veranstaltungen aufgetreten, aber heute treffen wir uns eher, um nur so zum Spaß zu spielen. Zwar ist aus mir leider kein Profi-Musiker geworden, aber ich bin Musikjournalist und schreibe – natürlich – am liebsten über basslastige Musik. Aber es geht bei mir auch nicht immer nur um Musik. Seit diesem Jahr bin ich Hobby-Imker und halte auf meinem Balkon Bienen. Außerdem versuche ich gerade Italienisch zu lernen. Ich glaube, man könnte also sagen, dass ich recht vielseitige Interessen habe. Aber die Bassgitarre begleitet mich eben schon am längsten.

I've been playing bass guitar since I was a teenager. I used to perform regularly at events with my band, but nowadays we tend to get together to play just for fun. Unfortunately, I didn't become a professional musician. Anyhow, I am a music journalist and – of course – I prefer to write about bass-heavy music. But it's not

always just about music for me either. Since this year I am a hobby beekeeper and keep bees on my balcony. I'm also trying to learn Italian right now. So, I guess you could say that I have quite diverse interests. Nevertheless, the bass guitar has accompanied me for the longest time.

Expertise does not match the critical object (bass guitar):

Ich spiele seit meiner Jugend Basketball. Früher bin ich mit meiner Mannschaft regelmäßig bei Turnieren angetreten, aber heute treffen wir uns eher, um nur so zum Spaß zu spielen. Zwar ist aus mir leider kein Profi-Basketballer geworden, aber ich bin Sportjournalist und schreibe – natürlich – am liebsten über Basketball. Aber es geht bei mir auch nicht immer nur um Sport. Seit diesem Jahr bin ich Hobby- Imker und halte auf meinem Balkon Bienen. Außerdem versuche ich gerade Italienisch zu lernen. Ich glaube, man könnte also sagen, dass ich recht vielseitige Interessen habe. Aber der Basketball begleitet mich eben schon am längsten.

I've been playing basketball since I was a teenager. I used to compete regularly in tournaments with my team, but nowadays we tend to get together to play just for fun. Unfortunately, I didn't become a professional basketball player. Anyhow, I am a sports journalist and – of course – I prefer to write about basketball. But it's not always just about sports for me either. Since this year I am a hobby beekeeper and keep bees on my balcony. I'm also trying to learn Italian right now. So, I guess you could say that I have quite diverse interests. Nevertheless, basketball has accompanied me for the longest time.

Figure 4

Example Stimulus From Study 3

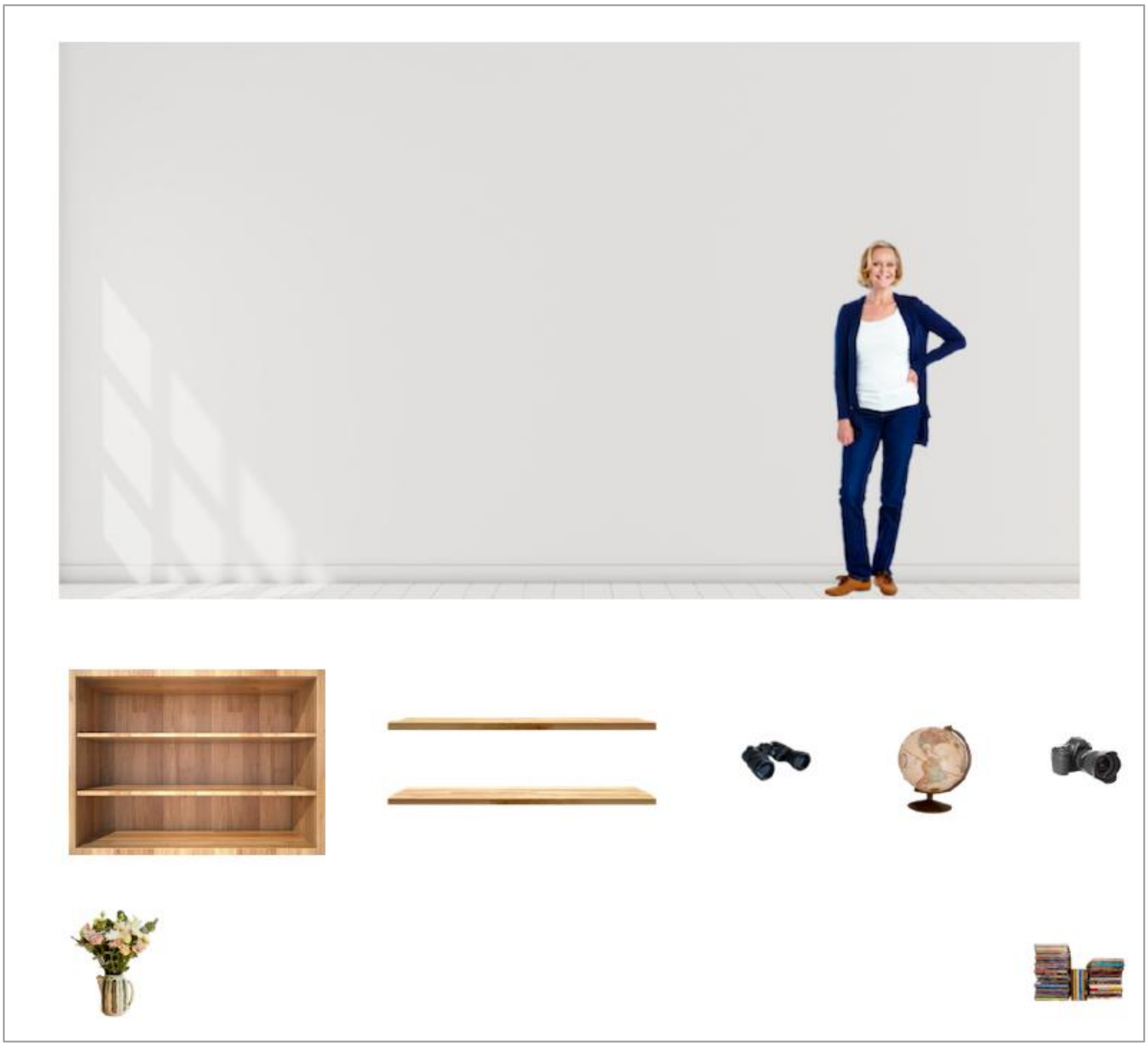

Note. The illustration depicts one of three trials. Depending on the condition, the critical object (in this case, the camera) matched the target's previously described expertise (photography) or did not (pastry). Participants' task was to place the objects in the room as they believed the target would.

As a post-hoc analysis, we computed an independent *t*-test for each trial to compare the Euclidean distances between target persons and critical objects as a function of a match between the target's expertise and the critical objects. The results are displayed in Table 9.

Table 9

Averaged Euclidian Distances Between Target and Critical Object for Matching and Non-Matching Expertise

Critical objects	Expertise		$t(118)$	$p_{one\text{-}tailed}$	*Cohen's d*
	Matching	Non-matching			
Camera	335.58 (149.84)	380.30 (141.41)	-1.68	.048	.31
Dog	97.58 (34.26)	117.28 (62.12)	-2.16	.017	.39
Bass guitar	261.51 (153.73)	300.00 (181.31)	-1.26	.106	.23

Note. $N = 120$. The table displays averaged Euclidian distances between targets and critical objects depending on whether the target's expertise matched the critical object or not. The distances were measured in pixels, higher numbers represent greater distances. The standard deviations are in parentheses.

Moreover, we investigated the assumption that the expertise manipulation influenced where participants placed the critical and distractor objects differently. Therefore, we conducted a mixed ANOVA with expertise (matching vs. non-matching the critical object) as between–subjects factor and trials (Targets 1 to 3) as well as type of object (critical vs. distractor) as within–subjects variables. The analysis revealed a significant interaction between expertise and type of object, $F(1, 118) = 7.11$, $p = .009$, $\eta^2_p = .06$, and between trials and type of object, $F(2, 118) = 81.13$, $p < .001$, $\eta^2_p = .41$. Moreover, we found a significant main effect of trials, $F(2, 118) = 117.52$, $p <. 001$, $\eta^2_p = .50$ as well as of type of object, $F(2, 118) = 183.52$, $p < .001$, $\eta^2_p = .61$. Neither the three-way interaction between expertise, type of object, and trials, $F(2, 118) = 0.05$, $p = 954$, $\eta^2_p = 0$, nor the main effect of expertise reached statistical significance, $F(1, 118) = 3.49$, $p = .064$, $\eta^2_p = .03$.

The significant two-way interaction between expertise and type of object indicates that the target's expertise affected participants' placement of the two types of objects differently. Specifically, whether the critical object matched the target's expertise influenced where participants placed the critical, but not the distractor objects. The absence of a significant three-way interaction with trials suggests that the nuanced effect of expertise on the placement of both types of objects was independent of trial and target. The significant interaction between trials and type of object implies that the distance at which participants placed both the critical and distractor objects from the targets varied significantly across trials.

Study 4

Below, the reader will find the stimulus material we used in Study 4.

Figure 5

Stimulus Material Employed in Study 4

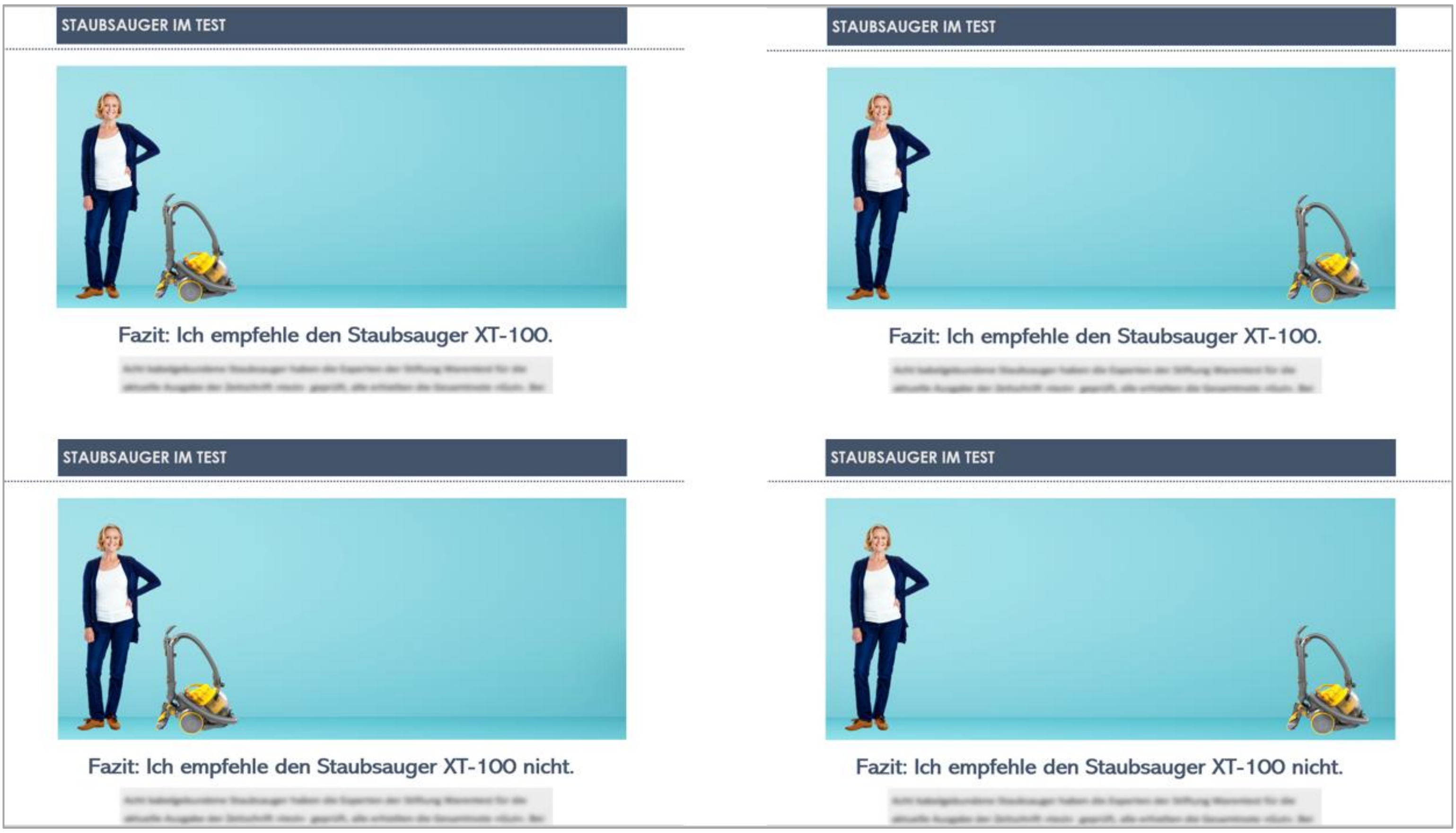

Note. The figure displays the stimuli used in Study 4. The self-designed images depicted the female communicator at either a short or large spatial distance from the attitude object (a vacuum cleaner). Beneath the images, participants read a message presenting advice either in favor of or against the vacuum cleaner, depending on condition. The blurred text lines below were intended to simulate a paywalled website containing detailed test reports.

Moderated Mediation für Liking as Mediator.

Neither the index of moderated mediation, $b = -.02$, 95% BootCI [−0.25, 0.21], nor the interaction between spatial distance and advice in predicting liking (the a-path) was significant, $b = -.09$, $p = .845$, 95% BootCI [−1.01, 0.83]. The effect of distance on liking was also not significant, $b = -0.41$, $p = .216$, 95% CI [−1.06, 0.24]. In contrast, the effect of liking on attitude (b-path) was significant, $b = 0.23$, $p = .002$, 95% CI [0.09, 0.38], as was the interaction between distance and advice in predicting attitude (c′-path), $b = 0.81$, $p = .028$, 95% CI [0.09, 1.53] (Figure 6). Conditional direct effects were consistent with opposite directional patterns, with spatial distance being negatively related to attitudes when advice was in favor of the object ($b = -0.34$, $p = .195$) and positively related when advice was against the object ($b = 0.47$, $p = .070$), although neither effect reached statistical significance.

Figure 6

Conditional Indirect Effects of Spatial Distance on Attitude via Liking

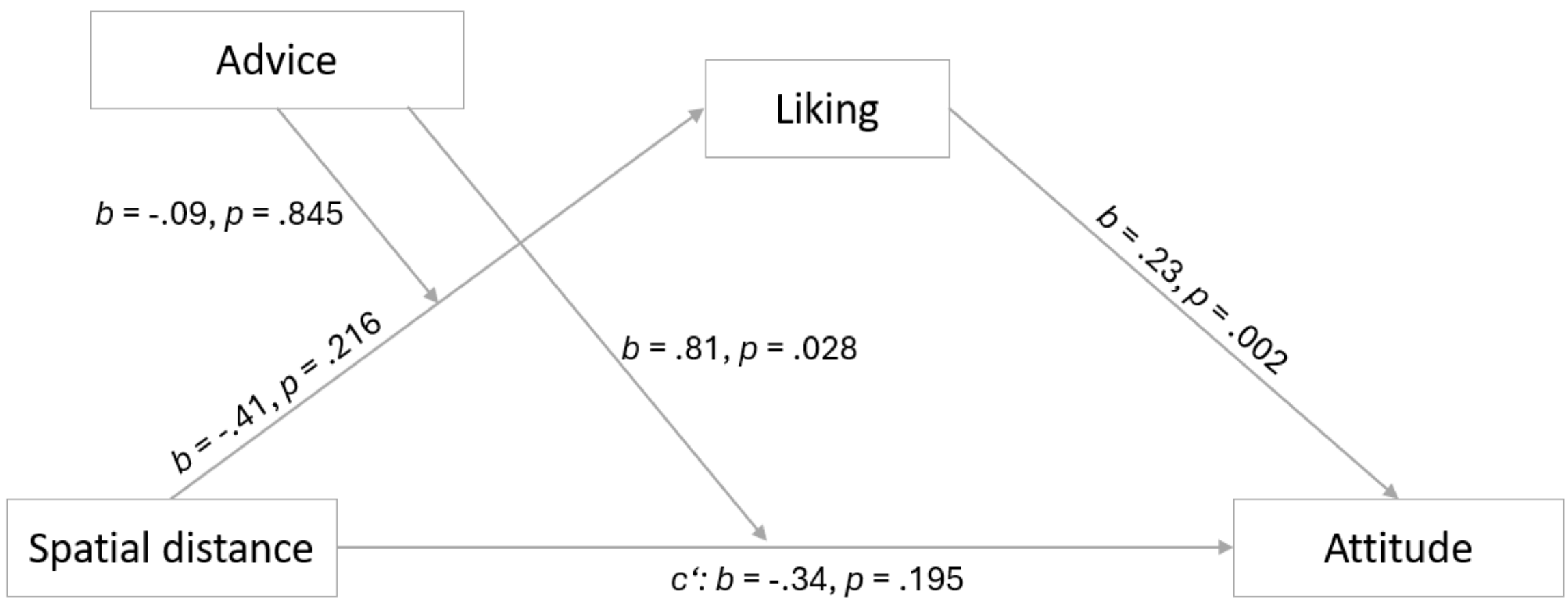

Note. $N = 119$. Unstandardized indirect effects are reported. Bootstrap confidence intervals are based on 5,000 resamples. Distance was coded 0 = proximal and 1 = distal; Advice was coded 0 = in favor and 1 = against.
Model for the a-path $R^2 = .03$, $F(3,115) = 1.29$, $p = .283$. Model for b-path and c’- $R^2 = .17$, $p = .028$.

Table 10

Results of the Factorial ANOVA for Liking

Variable	Type III Sum of Squares	*df*	Mean Square	*F*	*p*	η^2_p
(Intercept)	1841.83	1	1841.83	1160.46	< .001	.91
Distance	6.06	1	6.06	3.82	.053	.03
Advice	.01	1	.01	.01	.928	0
Distance × Advice	.06	1	.06	.04	.845	0

Note. *N* = 119. The table shows the results of a univariate ANOVA on liking with spatial distance (proximal vs. distal) and advice (in favor vs. against) as between–subjects factors.
